# The Protective Effects of Curcumin on Obesity-Related Glomerulopathy Are Associated with Inhibition of Wnt/*β*-Catenin Signaling Activation in Podocytes

**DOI:** 10.1155/2015/827472

**Published:** 2015-10-11

**Authors:** Bao-li Liu, Yi-pu Chen, Hong Cheng, Yan-yan Wang, Hong-liang Rui, Min Yang, Hong-rui Dong, Dan-nuo Han, Jing Dong

**Affiliations:** ^1^Division of Nephrology, Beijing Anzhen Hospital, Capital Medical University, Beijing 100029, China; ^2^Division of Nephrology, Beijing Traditional Chinese Medicine Hospital, Capital Medical University, Beijing 100010, China

## Abstract

The present study investigated the effects of curcumin, one of the most important active ingredients of turmeric, on podocyte injury *in vitro* and obesity-related glomerulopathy (ORG) *in vivo*. Cellular experiments *in vitro* showed that curcumin significantly antagonized leptin-induced downregulation of the mRNA and protein expression of podocyte-associated molecules including nephrin, podocin, podoplanin, and podocalyxin. Animal experiments *in vivo* showed that curcumin significantly reduced the body weight, Lee's index, abdominal fat index, urinary protein excretion, and average glomerular diameter and significantly upregulated the mRNA and protein expressions of the above podocyte-associated molecules in ORG mice. Furthermore, the experiments *in vitro* and *in vivo* both displayed that curcumin could downregulate the mRNA and protein expressions of Wnt1, Wnt2b, Wnt6, and *β*-catenin and upregulate the phosphorylation level of *β*-catenin protein in podocytes and renal tissue. In conclusion, curcumin is able to alleviate the harmful reaction of leptin on podocytes and reduce the severity of ORG. The above protective effects are associated with the inhibition of Wnt/*β*-catenin signaling activation in podocytes.

## 1. Introduction

In the past two decades, the obese patients were obviously increased with the improvement of life conditions in China. Obesity is a risk factor not only for diabetes mellitus and cardiovascular diseases, but also for kidney disease, which can induce obesity-related glomerulopathy (ORG) [1, 2]. As early as 1974, Weisinger and colleagues [3] firstly found that the severely obese individuals might be associated with massive proteinuria. With a rise in the number of obese patients, the incidence of ORG was rapidly increased. Kambham and colleagues [4] reported that among 6818 patients who underwent renal biopsy, the percentage of ORG patients increased from 0.2% in 1986–1990 to 2% in 1996–2000. Cheng and Chen [5] reported that ORG patients accounted for 3.8% in 1186 cases of renal biopsy during 2006–2008. Progression of ORG is relatively slow, but finally it still can enter into end stage renal disease (ESRD). Therefore, prevention and treatment of ORG have attracted more and more attention [1, 2].

Unfortunately except restricting dietary caloric intake, appropriately increasing physical activity and taking insulin sensitizing agent, so far there are scarcely other measures that can effectively interfere with ORG. So it is very important to look for available medicines. Curcumin, one of the most important active ingredients of turmeric, is a possible candidate. The modern laboratory and clinical research work in China and other countries has showed that curcumin is able to promote body weight loss and reduce incidence of some obesity-related diseases, such as diabetes mellitus, ischemic heart disease, stroke, and cancer [6–9]. In the present study, we are going to investigate the effects of curcumin on prevention and treatment of ORG and to explore its possible signal transduction pathway by the use of cellular experiments* in vitro* and animal experiments* in vivo*.

## 2. Materials and Methods

### 2.1. Cellular Experiments

#### 2.1.1. Cell Culture

The conditionally immortalized mouse podocyte cell line was kindly provided by Proffessor Maria Pia Rastaldi (S. Carlo Hospital, University of Milan). Cells were incubated in RPMI-1640 medium (Gibco) containing 10% inactivated fetal bovine serum (FBS) and 10 u/mL interferon-*γ* (IFN-*γ*) (Cell Signaling Technology) at 33°C in humidified air with 5% CO_2_. When cells reached 70% to 80% confluence, the cells were transferred in RPMI-1640 medium containing 10% inactivated FBS without IFN-*γ* and incubated at 37°C with 5% CO_2_ to induce differentiation. The cells would completely differentiate in 10 to 14 days.

#### 2.1.2. Immunofluorescent Staining of Nephrin and Synaptopodin on Podocytes

**Figure 1:**
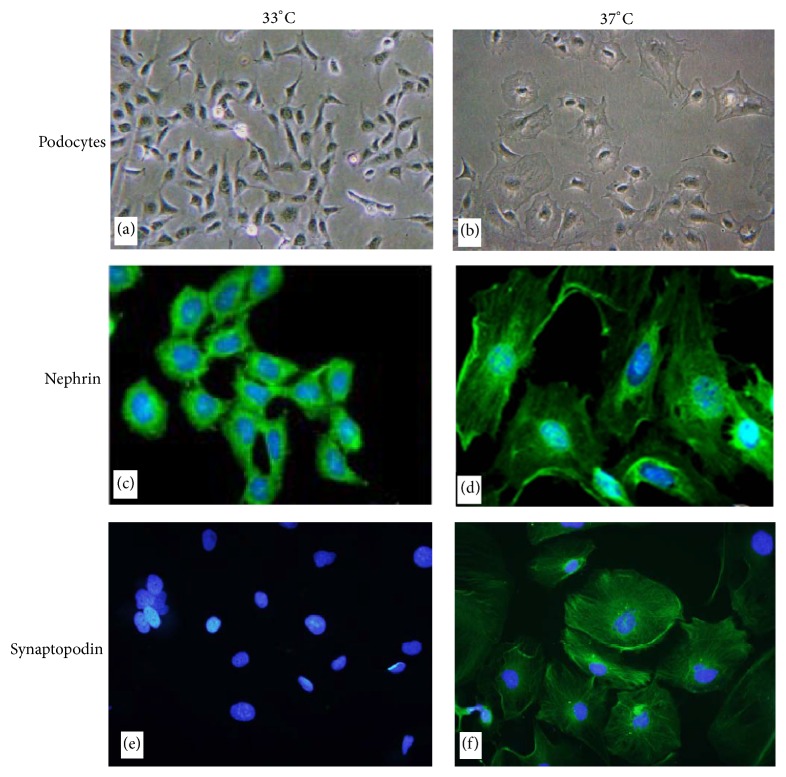
Cultured conditionally immortalized mouse podocytes in undifferentiated and differentiated statuses. (a) and (b) Morphology of cultured podocytes in undifferentiated status (left) and differentiated status (enlarged cell bodies with short and long projections, right) (phase microscopy ×200). (c) and (d) Diffuse cytoplasmic expression of nephrin in undifferentiated status (left); cell surface and cytoplasmic expression of nephrin in differentiated status (right) (immunofluorescence microscopy ×1000). (e) and (f) No expression of synaptopodin in undifferentiated status (left); filamentous and cell surface expression of synaptopodin in differentiated status (right) (immunofluorescence microscopy ×1000).

Before cellular experiments, differentiated status of podocytes was inspected by the indirect immunofluorescent staining of nephrin and synaptopodin, the marker proteins of podocytes [10]. Podocytes on the cover slip were fixed in 4% paraformaldehyde and permeabilized with 0.1% Triton X-100. Fixed podocytes were incubated overnight at 4°C with either rabbit anti-nephrin antibodies (1 : 100, Abcam) or mouse anti-synaptopodin antibodies (1 : 100, Acris GmbH). After washing with PBS for 3 times, podocytes were incubated with FITC-labeled goat anti-rabbit IgG antibodies (Dako) or FITC-labeled goat anti-mouse IgG antibodies (Dako) for 1 h at room temperature, mounted with a Hochst33342 containing mounting solution, and then observed with a fluorescent microscope (Nikon, Japan). The images of undifferentiated and differentiated podocytes were shown in Figure 1.

#### 2.1.3. Effects of Leptin and Curcumin on the Podocytes

The podocytes were incubated in RPMI-1640 medium, medium containing 15 ng/mL leptin (Abcam), medium containing 5 *μ*mol/L curcumin (Sigma-Aldrich), or medium containing both 15 ng/mL leptin and 5 *μ*mol/L curcumin, respectively. Curcumin 3.684 mg was dissolved in dimethyl sulphoxide (DMSO) 1 mL and then diluted into concentration of 5 *μ*mol/L with RPMI-1640 medium for experiment. After 9 h and 24 h of incubation, the mRNA and protein expression of nephrin, podocin, podoplanin, and podocalyxin of podocytes were determined by real-time PCR and Western blot, respectively. After 8 hours of incubation, the protein expression of phosphorylated *β*-catenin and total *β*-catenin of podocytes was measured by Western blot.

#### 2.1.4. Effects of Wnt Signaling Inhibitor DKK1 on the Podocytes

The podocytes were incubated in RPMI-1640 medium, medium containing 15 ng/mL leptin (Abcam), medium containing 200 ng/mL DKK1, that is, Dickkopf-1 (R&D), or medium containing both 15 ng/mL leptin and 200 ng/mL DKK1, respectively. Incubation time and observation items were the same as in Section 2.1.3.

### 2.2. Animal Experiments

#### 2.2.1. Animal Model and Grouping

Twenty-one male 5-week-old C57BL/6J mice were randomly divided into the following three groups: control group, ORG model group, and curcumin intervention group. The mice in control group were fed with common food which contains fat accounting for 10% kcal. The mice in model group and curcumin group were fed with high fat diet which contains fat accounting for 60% kcal according to the method described by us previously [11]. From 8th week to 12th week, the mice in curcumin group were subcutaneously injected 150 mg/kg/d curcumin that was dissolved in 75% polyethylene glycol 400 to a final concentration of 30 mg/mL. The same dosage of polyethylene glycol 400 was given to the mice in control and model groups in the same time. All the mice were sacrificed at the end of 12th week. A part of kidney tissue was fixed in 4% neutral formaldehyde solution for light microscopy, and another part of kidney tissue was rapidly preserved in liquid nitrogen for real-time PCR and Western blot assays.

#### 2.2.2. Physicochemical Parameters

Body weight was measured every week and body length was measured at the 12th week. The samples of nocturnal 12 h urine were collected at 0 week and 12th week, respectively, for the detection of urinary protein excretion. Blood and urine samples were collected at the 12th week to test serum cholesterol, blood glucose, and blood and urine creatinine levels (Olympus AU5400). The weight of visceral fat mass was measured after the mouse was sacrificed.

The calculation formulas were as follows: Lee's index = [body weight (g) × 1000]^1/3^ ÷ body length (cm); visceral fat index = visceral fat mass (g) ÷ body weight (g) × 100%; creatinine clearance rate (CCr) = urine creatinine × urine volume ÷ serum creatinine (mL/min).

#### 2.2.3. Glomerular Diameter Measurement

The tissue of kidney cortex was conventionally dehydrated, embedded, cut into sections, and stained with periodic acid-Schiff reagent. Twenty images of glomerular maximal profiles with vascular pole and/or urinary pole were taken under high-power microscope and were then analyzed by Nikon NIS-Elements BR image analysis software. The two longest perpendicular diameters of every glomerular capillary tuft without Bowman's space were measured and their mean value was calculated [11].

### 2.3. RNA Extraction and Real-Time PCR

Total RNA of each sample was extracted using TRIzol reagent (Invitrogen) according to the manufacturer's description. 2 *μ*g mRNA from each sample was reverse-transcribed to cDNA with Moloney murine leukemia virus reverse transcriptase (Transgene). Gene-specific primers were designed and synthesized by SBS Genetech Co., Ltd (Table 1). Relatively quantitative real-time PCR was performed in a total volume of 50 *μ*L containing 2 *μ*L of cDNA, 0.2 *μ*M each primer, and 25 *μ*L SYBR Green I Real-time PCR Master Mix (Transgene) using Rotor-Gene 6000. A thermal cycling profile consisted of a preincubation step at 94°C for 5 min, followed by 50 cycles of denaturation (94°C, 15 s), annealing (58°C, 15 s), and extension (72°C, 45 s). The specificity of the primers was verified by melting curves, and amplified products were sequenced to ensure the validity. Reactions were performed in triplicate, and threshold cycle numbers were averaged. A no template control was used as a negative control. The GAPDH fragment was amplified as a reference gene. The relative quantity of mRNA expression was calculated according to the formula 2^−(target  gene  Ct−GAPDH  Ct)^ × 10^3^, where Ct is the threshold cycle number [12].

### 2.4. Western Blot Assay

Total protein was extracted from the cultured podocytes or mouse renal cortex using cell lysis buffer. Protein samples were boiled for 5 minutes, separated by 10% sodium dodecyl sulphate-polyacrylamide gel electrophoresis (SDS-PAGE), and then transferred to nitrocellulose membranes (Amersham Pharmacia Biotech). After blocking with 5% skim milk in phosphate-buffered saline with 0.1% Tween 20 for 1 hour, the membranes were incubated with primary antibody at 4°C overnight and then incubated with secondary antibody in room temperature for 1 hour. Details regarding primary and secondary antibodies are listed in Table 2. The blotted proteins were quantified using Odyssey infrared imaging system. *β*-actin as internal control was used to assess equal loading and the relative protein level was expressed as the protein/*β*-actin ratio [13]. All assays were performed at least in triplicate.

### 2.5. Statistical Analysis

All of the data of continuous variables were represented as mean ± SD and analyzed by using SPSS 19.0 statistical software. One-way ANOVA was used to test the differences among groups. Paired *t*-test was used for matched-pairs samples.

## 3. Results

### 3.1. Curcumin Can Reduce Podocyte Injury Induced by Leptin

**Figure 2:**
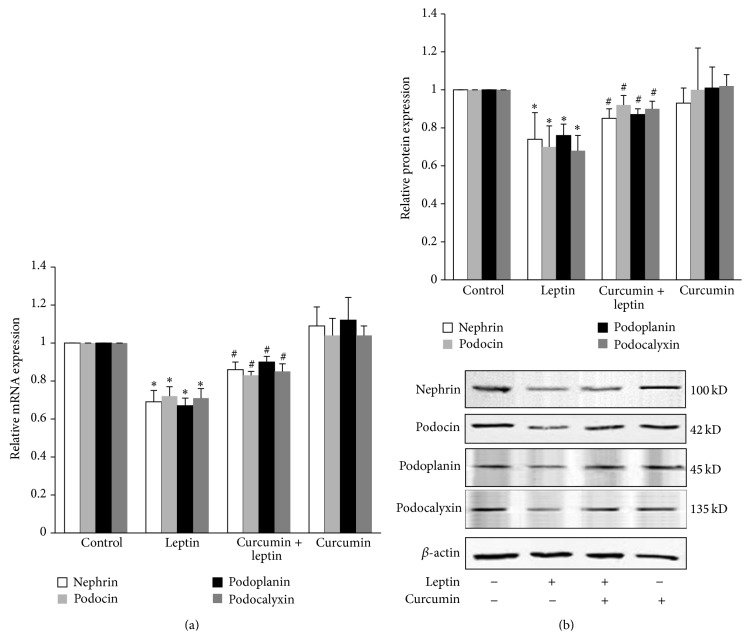
Effects of curcumin on leptin-mediated podocyte injury. Well-differentiated conditionally immortalized mouse podocytes were incubated in normal medium, medium containing 15 ng/mL leptin, medium containing 5 *μ*mol/L curcumin, or medium containing both 15 ng/mL leptin and 5 *μ*mol/L curcumin, respectively. (a) After 9 h, cells were collected and the relative mRNA expression levels of nephrin, podocin, podoplanin, and podocalyxin were measured by relative quantitative real-time RT-PCR. (b) After 24 h, cells were lysed and total lysates were analyzed by Western blot assay with antibodies of nephrin, podocin, podoplanin, podocalyxin, and *β*-actin, respectively. The relative protein level was expressed as the protein/*β*-actin ratio. Values are represented as mean ± SD (*n* = 3). ^∗^ *P* < 0.05 versus control group and ^#^ *P* < 0.05 versus leptin alone group.

*In vitro* cellular experiments showed that, compared with control group, leptin significantly downregulated the mRNA and protein expression of podocyte-associated molecules including nephrin, podocin, podoplanin, and podocalyxin (*P* < 0.05), while curcumin had no effect on their expression (*P* > 0.05). Compared with leptin group, curcumin significantly upregulated the mRNA and protein expression of above podocyte-associated molecules (*P* < 0.05) (Figure 2).

### 3.2. Curcumin Can Relieve Renal Damage in ORG Mice

#### 3.2.1. Effects of Curcumin on the Physicochemical Parameters of Mice

**Figure 3:**
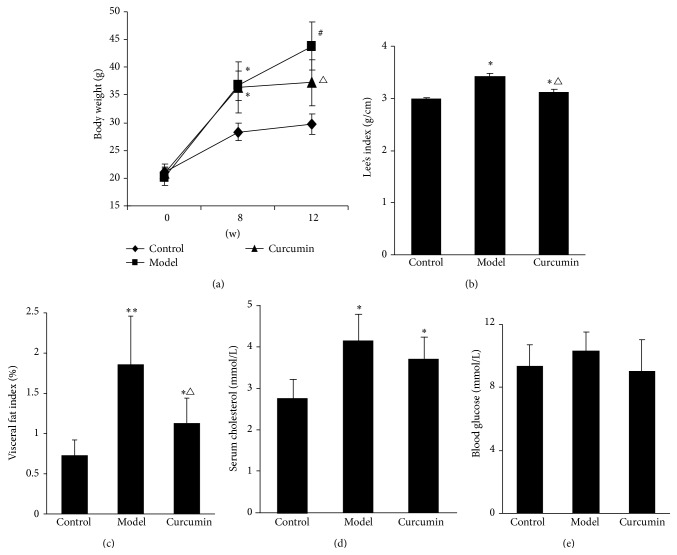
Effects of curcumin on body weight, Lee's index, visceral fat index, serum cholesterol, and blood glucose of ORG mice. (a) Average body weight of mice in model group and curcumin group was significantly higher than that in control group at the 8th week; it was significantly lighter in curcumin group than that in model group at the 12th week. ^∗^ *P* < 0.05 versus control group at 8th week, ^#^ *P* < 0.05 versus model group itself at 8th week, and ^△^ *P* < 0.05 versus model group at 12th week. (b), (c), and (d) Average Lee's index, visceral adiposity index, and serum cholesterol levels were significantly increased in mice of model group and curcumin group compared with control group, and the former two parameters were significantly reduced in curcumin group compared with model group at the 12th week. ^∗^ *P* < 0.05 versus control group, ^∗∗^ *P* < 0.01 versus control group, and ^△^ *P* < 0.05 versus model group. (e) Average levels of blood glucose in three groups had no significant difference at the 12th week. Values are represented as mean ± SD (*n* = 7).

*In vivo* animal experiments showed that, compared with control group, the body weight of mice in model group and curcumin group was significantly increased at the 8th week (*P* < 0.05). Compared with their own body weight at the 8th week, it was significantly increased again in model group (*P* < 0.05) but had no significant change in curcumin group at the 12th week (*P* > 0.05). In addition, the body weight of mice in curcumin group was significantly lighter than that in model group at the 12th week (*P* < 0.05) (Figure 3).

The average Lee's index and abdominal fat index of mice were significantly increased in model and curcumin groups compared with control group (*P* < 0.05 or *P* < 0.01), while these two parameters in curcumin group were significantly lower than those in model group at the 12th week (*P* < 0.05) (Figure 3).

At the 12th week, the average serum cholesterol levels of mice in model and curcumin groups were significantly increased compared with control group (*P* < 0.05). The absolute value of this parameter in curcumin group was lower than that in model group, but the difference had not reached statistical significance (*P* > 0.05). There was no significant difference of average blood glucose levels among the three groups (*P* > 0.05) (Figure 3).

#### 3.2.2. Effects of Curcumin on the Proteinuria and CCr of Mice

**Figure 4:**
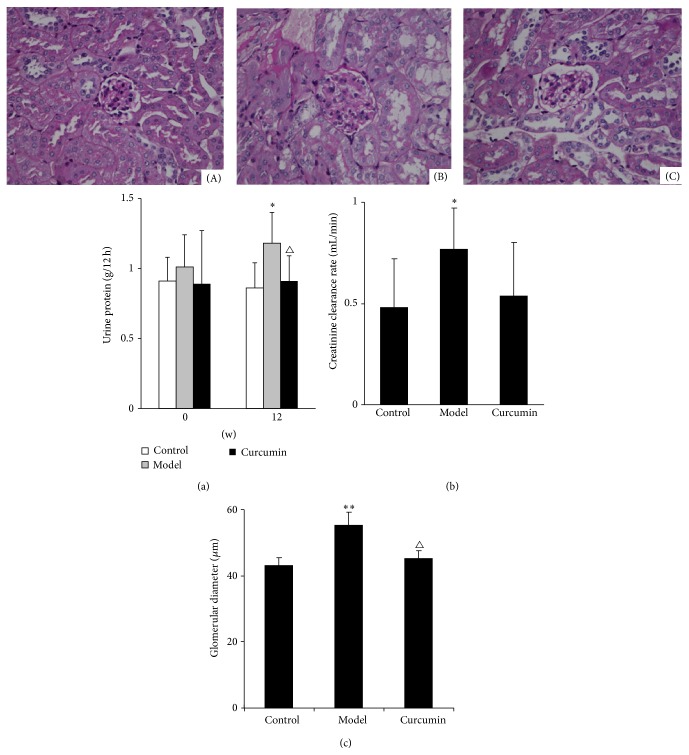
Effects of curcumin on renal damage of ORG mice. (a) Average level of nocturnal 12 h urine protein was significantly increased in mice of model group compared with control group and was significantly reduced in curcumin group compared with model group at the 12th week. (b) Average level of creatinine clearance rate was significantly increased in mice of model group compared with control group and was reduced in curcumin group compared with model, but the difference had not reached statistical significance at the 12th week. (c) Histology of kidney tissue from mice of control (A), model (B), and curcumin (C) groups (PAS staining ×400). The average glomerular diameter was significantly longer in the mice of model group than that in control group, while it was significantly shorter in curcumin group than that in model group at the 12th week. Values are represented as mean ± SD (*n* = 7). ^∗^ *P* < 0.05 versus control group, ^∗∗^ *P* < 0.01 versus control group, and ^△^ *P* < 0.05 versus model group.

There was no difference of the nocturnal 12 h urinary protein excretion among the three groups at the beginning of the experiment (*P* > 0.05). Compared with the beginning of the experiment, the nocturnal 12 h urinary protein excretion was significantly increased in model group (*P* < 0.05), but it had no significant change in curcumin or control groups at the 12th week (*P* > 0.05). In addition, the nocturnal 12 h urinary protein excretion in model group was significantly higher than that in control group (*P* < 0.05), while in the curcumin group it was significantly lower than that in model group at the 12th week (*P* < 0.05) (Figure 4).

At the 12th week, the average CCr of mice in model group was significantly increased compared with control group (*P* < 0.05). The absolute value of CCr in curcumin group was lower than that in model group, but the difference had not reached statistical significance (*P* > 0.05) (Figure 4).

#### 3.2.3. Effect of Curcumin on Glomerular Size of Mice

The average glomerular size, which was expressed as average glomerular diameter in the present study, was significantly larger in the model group than that in control group (*P* < 0.01), while it was significantly smaller in curcumin group than that in model group at the 12th week (*P* < 0.05) (Figure 4).

#### 3.2.4. Effects of Curcumin on Podocyte-Associated Molecules of Mice

**Figure 5:**
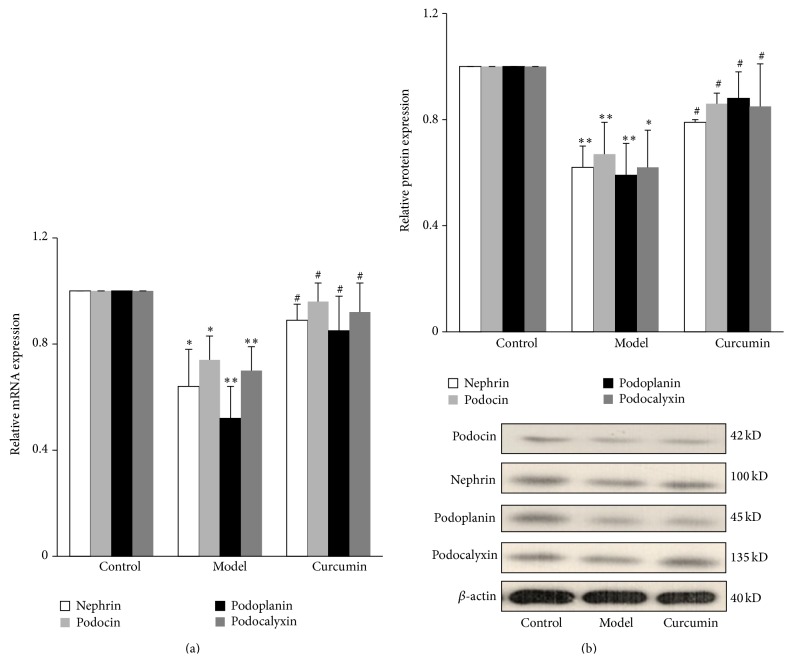
Effects of curcumin on podocyte-associated molecules of mice. (a) Mice sacrificed at the end of 12th week. Total RNA was extracted from kidney cortex, and then the relative mRNA expression levels of nephrin, podocin, podoplanin, and podocalyxin were measured by relative quantitative real-time RT-PCR. (b) Kidney cortex tissue was lysed and total lysates were analyzed by Western blot assay with antibodies of nephrin, podocin, podoplanin, podocalyxin, and *β*-actin antibody, respectively. The relative protein level was expressed as the protein/*β*-actin ratio. Values are represented as mean ± SD (*n* = 7). ^∗^ *P* < 0.05 versus control group, ^∗∗^ *P* < 0.01 versus control group, and ^#^ *P* < 0.05 versus model group.

Compared with control group, the mRNA and protein expression of podocyte-associated molecules, including nephrin, podocin, podoplanin, and podocalyxin, were significantly decreased in the renal cortical tissue of mice in model group at the 12th week (*P* < 0.05 or *P* < 0.01). Compared with model group, the mRNA and protein expression of the above podocyte-associated molecule were significantly upregulated in curcumin group at the 12th week (*P* < 0.05) (Figure 5).

### 3.3. Curcumin Can Inhibit the Wnt/*β*-Catenin Signaling Activation in Podocytes

#### 3.3.1. Inhibitory Effects of Curcumin on Podocyte Wnt/*β*-Catenin Signaling

**Figure 6:**
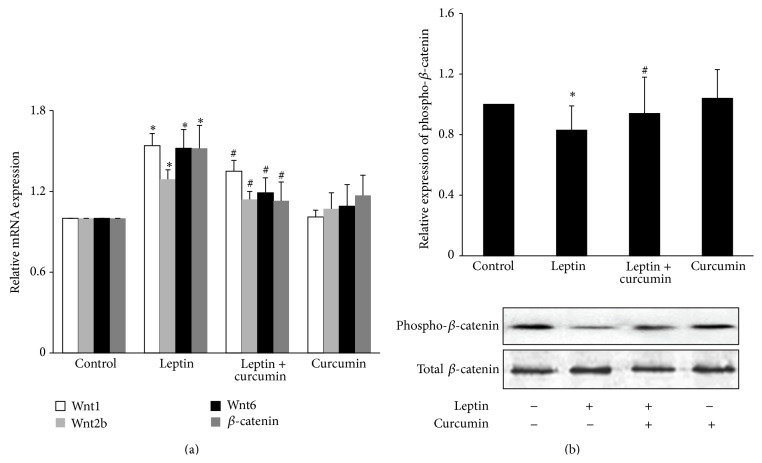
Effects of curcumin on leptin-activated Wnt/*β*-catenin signaling in cultured podocytes. Well-differentiated conditionally immortalized mouse podocytes were incubated in normal medium, medium containing 15 ng/mL leptin, medium containing 5 *μ*mol/L curcumin, or medium containing both 15 ng/mL leptin and 5 *μ*mol/L curcumin, respectively. (a) After 9 h, cells were collected and the mRNA expression levels of Wnt1, Wnt2b, Wnt6, and *β*-catenin were measured by relative quantitative real-time RT-PCR. (b) After 24 h, cells were lysed and total lysates were analyzed by Western blot assay with anti-phosphorylated *β*-catenin antibody and total *β*-catenin antibody, respectively. The relative phosphorylated *β*-catenin level was expressed as the phosphorylated *β*-catenin/total *β*-catenin ratio. Values are represented as mean ± SD (*n* = 3). ^∗^ *P* < 0.05 versus control group and ^#^ *P* < 0.05 versus leptin alone group.

*In vitro* cell experiments showed that, compared with control group, the mRNA expression of Wnt1, Wnt2b, Wnt6, and *β*-catenin was significantly upregulated (*P* < 0.05), and the level of phosphorylated *β*-catenin protein was significantly decreased in leptin group (*P* < 0.05). Curcumin alone had no effect on podocyte Wnt/*β*-catenin signaling pathway (*P* > 0.05) (Figure 6).

Compared with leptin group, the mRNA expression of the Wnt1, Wnt2b, Wnt6, and *β*-catenin was significantly downregulated (*P* < 0.05), and the level of phosphorylated *β*-catenin protein was significantly increased in curcumin group (*P* < 0.05) (Figure 6).

#### 3.3.2. Inhibitory Effects of Curcumin on Wnt/*β*-Catenin Signaling in the Kidney Cortex Tissue of ORG Mice

**Figure 7:**
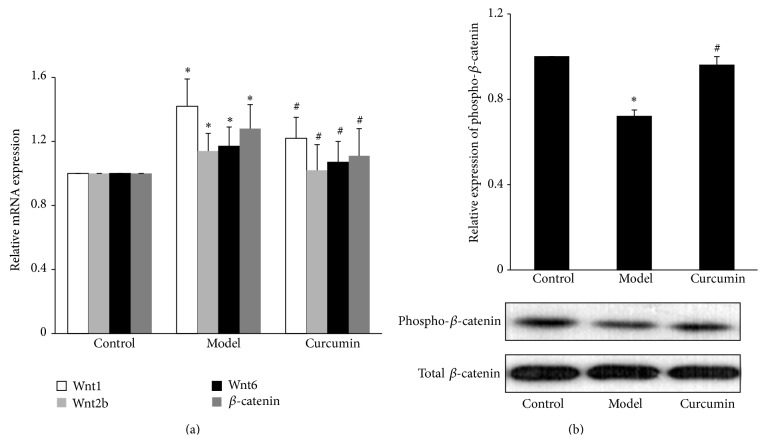
Effects of curcumin on Wnt/*β*-catenin signaling in the kidney tissue of ORG mice. (a) Mice sacrificed at the end of 12th week. Total RNA was extracted from kidney cortex, and then the relative mRNA expression levels of Wnt1, Wnt2b, Wnt6, and *β*-catenin were measured by relative quantitative real-time RT-PCR. (b) Kidney cortex was lysed and total lysates were analyzed by Western blot assay with anti-phosphorylated *β*-catenin antibody and total *β*-catenin antibody, respectively. The relative phosphorylated *β*-catenin level was expressed as the phosphorylated *β*-catenin/total *β*-catenin ratio. Values are represented as mean ± SD (*n* = 7). ^∗^ *P* < 0.05 versus control group and ^#^ *P* < 0.05 versus model group.

The mRNA expression of Wnt1, Wnt2b, Wnt6, and *β*-catenin was significantly upregulated in model group compared with control group (*P* < 0.05) and was significantly downregulated in curcumin group compared with model group at the 12th week (*P* < 0.05) (Figure 7).

At the 12th week, the level of phosphorylated *β*-catenin protein was significantly decreased in model group compared with control group (*P* < 0.05) and significantly increased in curcumin group compared with model group (*P* < 0.05) (Figure 7).

#### 3.3.3. Inhibition of Wnt/*β*-Catenin Signaling Activation Can Reduce Podocyte Injury

**Figure 8:**
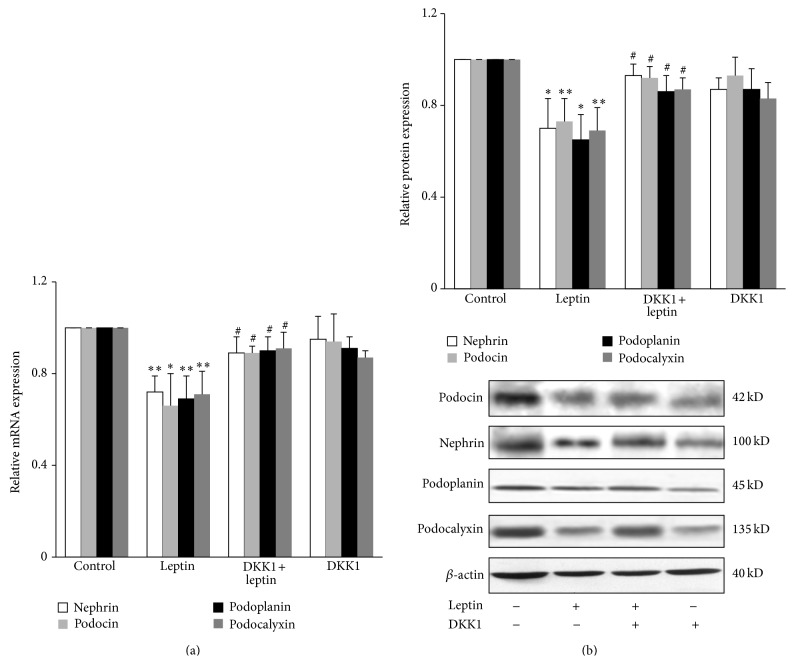
Effects of DKK1 on leptin-mediated podocyte injury. Well-differentiated conditionally immortalized mouse podocytes were incubated in normal media, media containing 15 ng/mL leptin and medium containing both 15 ng/mL leptin and 200 ng/mL DKK1, a well-known inhibitor of Wnt/*β*-catenin signaling, respectively. (a) After 9 h, cells were collected and the mRNA expression levels of nephrin, podocin, podoplanin, and podocalyxin were measured by relative quantitative real-time RT-PCR. (b) After 24 h, cells were lysed and total lysates were analyzed by Western blot assay with antibodies of nephrin, podocin, podoplanin, podocalyxin, and *β*-actin, respectively. The relative protein level was expressed as the protein/*β*-actin ratio. Values are represented as mean ± SD (*n* = 3). ^∗^ *P* < 0.05 versus control group, ^∗∗^ *P* < 0.01 versus control group, and ^#^ *P* < 0.05 versus leptin alone group.

To test whether Wnt/*β*-catenin signaling activation was involved in the podocyte injury induced by leptin, the inhibitor of Wnt/*β*-catenin signaling DKK1 was used for cellular experiments. Result showed that the mRNA and protein expression of nephrin, podocin, podoplanin, and podocalyxin in leptin group were significantly downregulated compared with control group (*P* < 0.05 or *P* < 0.01), while DKK1 group was significantly upregulated compared with leptin group (*P* < 0.05) (Figure 8).

## 4. Discussion

Turmeric belongs to plants of ginger family. Its rhizome is used as a traditional Chinese herb. Turmeric contains a wide variety of phytochemicals in which curcumin is the most important pharmacodynamic ingredient and makes up about 2–5% of turmeric [14]. Curcumin is a natural polyphenolic compound with a molecular formula of C_21_H_20_O_6_ [8, 14]. For investigating the action of curcumin on ORG and podocyte injury, it was used in cellular and animal experiments in the present study.

Podocyte injury and dysfunction are hallmark of ORG. Pathological examination in the kidney tissue of patients with ORG showed podocyte swelling, hypertrophy and vacuolar degeneration, increase of foot process width, fusion of foot process, decrease of podocyte density and number, the stripping of podocyte from the basement membrane, and so forth. Furthermore, these podocyte injuries were closely associated with proteinuria and renal function damage [15, 16]. In addition, it has been known that obese patients often have hyperleptinemia and leptin may play a role in the genesis of ORG [17, 18]. Therefore, in the* in vitro* cellular experiments of this study mouse podocytes were incubated with leptin, accompanied with curcumin or not, to observe the changes of podocytes. Results showed that leptin downregulated the mRNA and protein expression of podocyte-associated molecules including nephrin, podocin, podoplanin, and podocalyxin. The above podocyte-associated molecules are located at the slit diaphragm, basolateral region, basal aspect, and apical part of foot process, respectively, and have very important role in maintaining normal podocyte architecture and glomerular filtration function. The downregulation of their mRNA and protein expressions indicates podocyte injury [19]. However, our study results showed that curcumin could antagonize the harmful action of leptin and relieve the podocyte injury that resulted from leptin.

On the basis of cellular experiments, we carried out* in vivo* animal experiments using the mouse ORG model which was successfully established by our research team [11]. Study results showed that, compared with model group, the body weight, Lee's index, and abdominal fat index of mice in curcumin intervention group were significantly decreased, which suggests that curcumin has an effect against obesity including abdominal obesity. Study results also showed that the mice in curcumin intervention group had less urinary protein excretion, less severe glomerulomegaly, and enhanced mRNA and protein expressions of podocyte-associated molecules compared with model group, which suggests that curcumin also has a good effect on prevention and treatment of ORG.

It is reported that Wnt/*β*-catenin signaling in podocytes plays a critical role in integrating cell adhesion, motility, differentiation and survival [20], and Wnt/*β*-catenin signaling activation-mediated podocyte injury and proteinuria caused by puromycin, adriamycin, or TGF-*β* [21–23]. So, the present study investigated the effects of curcumin on Wnt/*β*-catenin signaling pathway in podocytes. The results of* in vitro* cellular experiments and* in vivo* animal experiments both showed that curcumin was able to downregulate the high expression of Wnt1, Wnt2b, Wnt6, and *β*-catenin and to raise the low phosphorylation level of *β*-catenin protein, which suggests that curcumin has an inhibition action on Wnt/*β*-catenin signaling activation in podocytes. In order to better understand the effect of inhibiting Wnt/*β*-catenin signaling on podocytes, the inhibitor of Wnt signaling DKK1 was used in the cellular experiment. Experimental result showed that inhibition of Wnt/*β*-catenin signaling did alleviate podocyte injury. So, we consider that the inhibition action of curcumin on Wnt/*β*-catenin signaling probably is one of its important mechanisms for prevention and treatment of ORG.

However, when Ahn and colleagues [24] studied the action of curcumin on 3T3-L1 preadipocytes, they found that curcumin was able to activate Wnt/*β*-catenin signaling, and therefore the differentiation and maturation of preadipocytes were suppressed. So, we speculate that curcumin may have different effects on the Wnt signaling pathway in different cells, which is quite worth a further study in the future. In addition, it has been known that curcumin can regulate multiple signaling pathways besides Wnt/*β*-catenin signaling [25], so is there another signaling pathway which also participates in the actions of curcumin on prevention and treatment of ORG? This is also worth our further research in the future.

## 5. Conclusion

Curcumin is able to alleviate the harmful reaction of leptin on podocytes and reduce the severity of ORG. The above protective effects are associated with the inhibition of Wnt/*β*-catenin signaling activation in podocytes. This study will provide a preliminary experimental basis to better use curcumin in prevention and treatment of ORG. To our knowledge, no similar study has been reported in the literature.

## Figures and Tables

**Table 1:** Sequence of oligo used in the study.

Name	Oligo sequence	Length of PCR products (bp)
Nephrin	Forward 5′-GTCTGGGGACCCCTCTATGA Reverse 5′-CAGGTCTTCTCCAAGGCTGT	209

Podocin	Forward 5′-CTGCAGAAGGGGAAAAGGCT Reverse 5′-TGATGCTCCCTTGTGCTCTG	205

Podoplanin	Forward 5′-AGGGAGGGACTATAGGCGTG Reverse 5′-GCTGAGGTGGACAGTTCCTC	202

Podocalyxin	Forward 5′-AGCCTGTGGATTCTTCACCG Reverse 5′-GTGTGGAGACGGGCAATGTA	210

Wnt1	Forward 5′-CGAACGACCGTGTTCTCTGA Reverse 5′-GCTCCAGGCGCAGCAG	191

Wnt2b	Forward 5′-GATGGGGCCAATTTCACAGC Reverse 5′-AGTTGTGTCATACCCTCGGC	202

Wnt6	Forward 5′-CAACTGGCTCTCCAGATGCT Reverse 5′-TGGCACTTACACTCGGTGC	203

*β*-catenin	Forward 5′-ACTGGAGCTCTCCACATCCT Reverse 5′-GTGGCTCCCTCAGCTTCAAT	191

GADPH	Forward 5′-TGTGAACGGATTTGGCCGTA Reverse 5′-GATGGGCTTCCCGTTGATGA-3′	206

**Table 2:** Primary and secondary antibodies used in the study.

Primary antibody	Secondary antibody
Rabbit anti-mouse nephrin pAb (Abcam)	IRDye 800 conjugated goat anti-rabbit IgG Ab (Rockland)

Rabbit anti-mouse podocin pAb (Sigma-Aldrich)	Ditto

Rabbit anti-mouse podoplanin pAb (Biotechnology)	Ditto

Rabbit anti-mouse podocalyxin pAb (Biotechnology)	Ditto

Rabbit anti-mouse *β*-catenin pAb (Cell Signaling Technology)	Ditto

Rabbit anti-mouse phosphorylated *β*-catenin pAb (Cell Signaling Technology)	Ditto

Mouse anti-mouse *β*-actin mAb (Sigma-Aldrich)	IRDye 800 conjugated goat anti-mouse IgG Ab (Rockland)

pAb: polyclonal antibody; mAb: monoclonal antibody.
